# Supplementary material for: Observers’ motivated sensitivity to stigmatized actors’ intent
Source: PLoS One. 2024 Sep 6;19(9):e0306119. doi: 10.1371/journal.pone.0306119 (PMC11379140; doi:10.1371/journal.pone.0306119)
Supplement: S1 File — (DOCX) [file pone.0306119.s001.docx]

**S1 Appendix**

**Both Proximal and Distal intent present**

- JG told his friends that he wanted to kill his rich uncle, as he stood to inherit a large sum of money. JG formulated a plan to kill his uncle at his uncle’s home by running him down with his car. JG began to drive speedily to his uncle’s home. As JG drove, he played over and over in his mind how he would spend the inheritance money. JG arrived at the house, saw his uncle in front, and intentionally pressed the accelerator. The car struck JG’s uncle, killing him instantly.

**Distal Intent High / Proximal Intent Low**

- JG told his friends that he wanted to kill his rich uncle, as he stood to inherit a large sum of money. JG formulated a plan to kill his uncle at his uncle’s home by running him down with his car. JG began to drive speedily to his uncle’s home. As JG drove, he played over and over in his mind how he would spend the inheritance money. All this thinking about his goal distracted JG from driving. As JG was going around a sharp turn near his uncle’s house, JG noticed a person crossing into the path of the car. Startled to see a person in the road, JG tried to press the brake but pressed the accelerator instead. The car struck the pedestrian, killing them instantly. The pedestrian turned out to be JG’s uncle.

**Proximal Intent High / Distal Intent Low**

- JG told his friends that he wanted to kill his rich uncle, as he stood to inherit a large sum of money. JG formulated a plan to kill his uncle at his uncle’s home by running him down with his car. JG began to drive speedily to his uncle’s home. As JG drove to his uncle’s home, JG calmed his nerves by focusing on his favorite song. As JG was going around a sharp turn near his uncle’s house, JG noticed a person crossing into the path of the car. Trying to get around the person in the road, JG intentionally pressed the accelerator. The car struck the pedestrian, killing them instantly. The pedestrian turned out to be JG’s uncle.

**Both Proximal Distal Intent Absent**

- JG told his friends that he wanted to kill his rich uncle, as he stood to inherit a large sum of money. JG formulated a plan to kill his uncle at his uncle’s home by running him down with his car. JG began to drive speedily to his uncle’s home. As JG drove to his uncle’s home, JG calmed his nerves by focusing on his favorite song. As JG was going around a sharp turn near his uncle’s house, JG noticed a person crossing into the path of the car. Startled to see a person in the road, JG tried to press the brake but pressed the accelerator instead. The car struck the pedestrian, killing them instantly. The pedestrian turned out to be JG’s uncle.

**S2 Appendix**

Politically neutral tweets (shown to both groups)


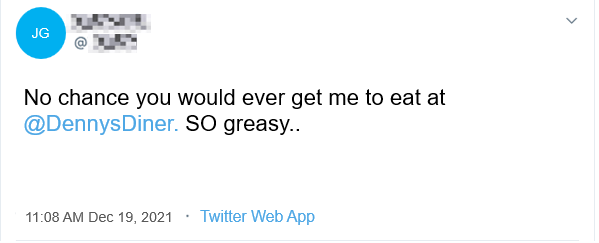

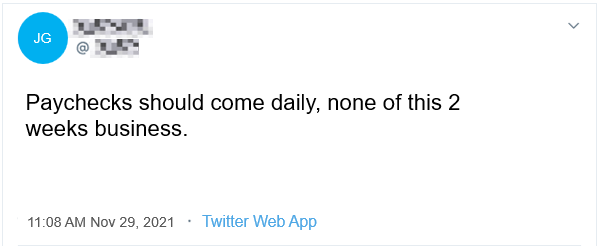


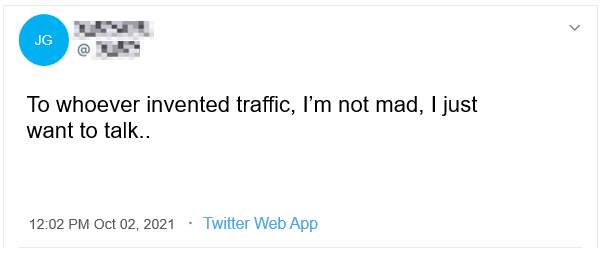

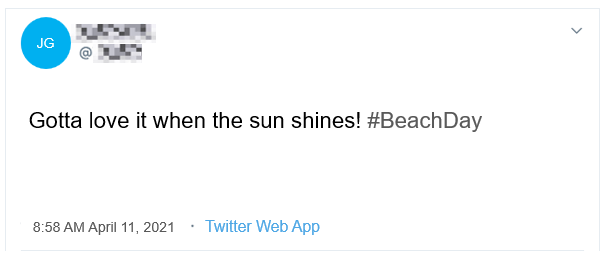


Politically left-aligned tweets (shown to participants selected to the ‘liberal actor’ condition)


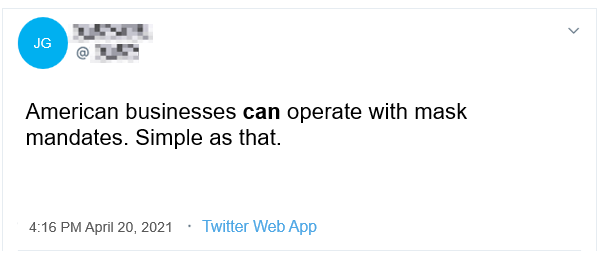

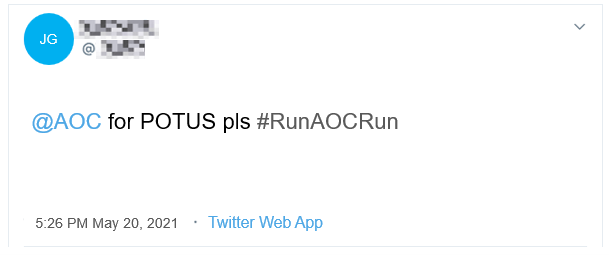


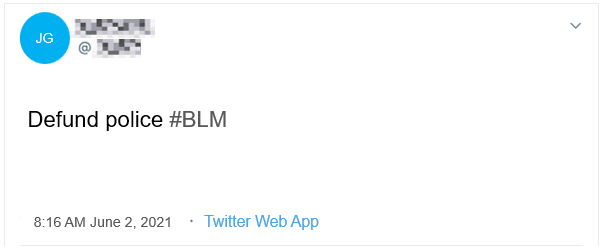

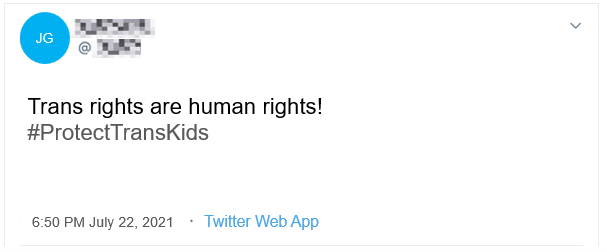


Politically right-aligned tweets (shown to participants selected to the ‘conservative actor’ condition)


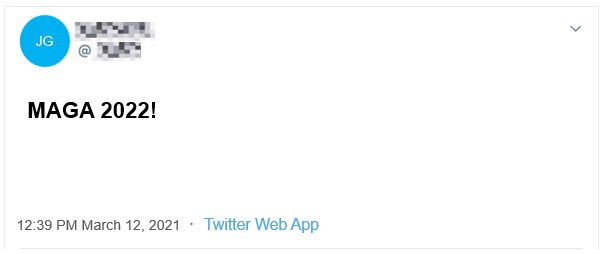

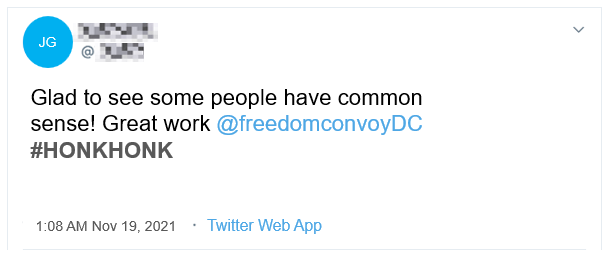


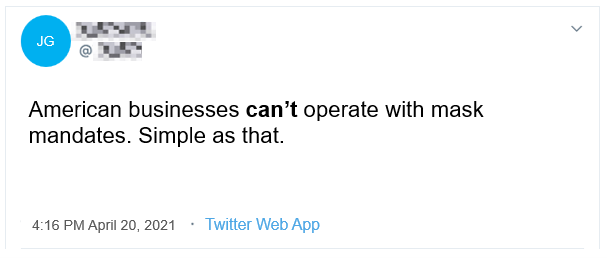

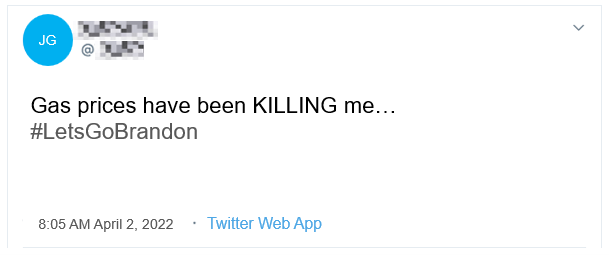


**S3 Appendix**

**Factor 1: Intent**

1. To what extent were JG’s actions intentional?
2. To what extent can one say that JG did what they did on purpose?

**Factor 2: Valence**

1. How positively should JG be judged?
2. How negatively should JG be judged?
3. How much blame should JG receive as a result of their actions?
4. Even if no one ever finds out what they did, JG will get what they deserve.

**S4 Appendix**

**Factor Analysis of MJI Items**

Factor 2 Factor 1 h2 u2 com

Item 1 0.51 0.86 0.9977 0.0023 1.6

Item 2 0.52 0.69 0.7496 0.2504 1.8

Item 3 0.02 -0.06 0.0037 0.9963 1.3

Item 4 0.78 0.07 0.6186 0.3814 1.0

Item 5 0.77 0.14 0.6182 0.3818 1.1

Item 6 0.02 0.03 0.0016 0.9984 1.9

- h2 = communality score (sum of the squared factor loadings for each item).
- u2 = uniqueness score.
- com = complexity

Factor 2 Factor 1

SS loadings 1.74 1.25

Proportion Variance 0.29 0.21

Cumulative Variance 0.29 0.50

Proportion Explained 0.58 0.42

Cumulative Proportion 0.58 1.00

- The degrees of freedom for the null model are 15 and the objective function was 2.24.
- The degrees of freedom for the model are 4 and the objective function was 0.03.
- The root mean square of the residuals (RMSR) is 0.03.
- The degrees of freedom corrected root mean square of the residuals is 0.06.

Factor 2 Factor 1

Correlation of (regression) scores with factors 0.87 0.96

Multiple R square of scores with factors 0.76 0.91

Minimum correlation of possible factor scores 0.51 0.83

**S5 Appendix**

**Skewness values:**

Full Moral Judgment Index: -0.885

Moral Judgment Index (Factor 1): -0.74

Moral Judgment Index (Factor 2): -1.556

**Skewness values following Square Transformation:**

Full Moral Judgment Index: -0.538

Moral Judgment Index (Factor 1): -0.338

Moral Judgment Index (Factor 2): - 1.146

**S6 Appendix**

**Attitude-Based Political Conservatism (ABPC) Scale:**

Please indicate your opinions on the following statements. If you are unsure how to answer any item, or would prefer not to answer, leave it blank.

| 1 | 2 | 3 | 4 | 5 | 6 | 7 |
| --- | --- | --- | --- | --- | --- | --- |
| Strongly Disagree | Disagree | Somewhat Disagree | Neither Agree nor Disagree | Somewhat Agree | Agree | Strongly Agree |

1. Proposed laws to reduce carbon emission are urgently needed and should be enacted immediately to save the planet from global warming.
2. The government should fund alternative energy research and production.
3. A large government is necessary to ensure that all our country’s citizens are taken care of.
4. Higher taxes (primarily for the wealthy) are necessary to address inequity/injustice in society.
5. The government should help the poor and needy using tax dollars from the rich.
6. The government should provide all citizens with free or low-cost health care.
7. Government programs are an important way to provide for the poor and needy in society.
8. The government should provide equal health care benefits for all, regardless of one’s ability to pay.
9. A smaller government with limited power would improve the standard of living for all.
10. The government should protect its citizens from the greed of big, private businesses.
11. Taxes enable the government to create jobs and provide welfare programs for those in need.
12. Health care should be completely privatized.
13. The government must produce a national plan for all energy resources with sustainability in mind.
14. The government has much more important things to fund than the health care system.
15. Health care in America should not be made public.
16. Government programs encourage people to become dependent and lazy, rather than encouraging work and independence.
17. I dislike the idea of tax dollars funding other people’s health care.
18. Homelessness is a problem that can only be solved with help from the government and tax dollars.
19. If someone wishes to die, he/she should have legal access to options for euthanasia.
20. A person has a right to die with dignity, by his/her own choice, therefore euthanasia should be legalized.
21. Euthanizing someone who wishes to die is murder.
22. Euthanasia of a human is wrong under all circumstances.
23. Abortion is murder.
24. If a pregnant woman believes abortion is the best choice for her, she should not have to defend that choice to anyone.
25. The use of embryonic stem cells in medicine could cause problems in the long run.
26. Even if someone requests medical euthanasia, it may not be the best solution for them.
27. Interracial marriages (i.e. a marriage between two people who are of different races) make me uncomfortable.
28. I wish that individuals of different races would not have children together.
29. I worry that people of different ethnicities having children will result in the disappearance of my own ethnicity.
30. I feel positive about people of different races having bi-racial children together.
31. Allowing refugees into America reduces the amount of resources available to American citizens.
32. I wish fewer American tax dollars were used for foreign aid.
33. It is easy for people from other countries to immigrate to America.
