# Supplementary material for: Observers’ motivated sensitivity to stigmatized actors’ intent
Source: PLoS One. 2024 Sep 6;19(9):e0306119. doi: 10.1371/journal.pone.0306119 (PMC11379140; doi:10.1371/journal.pone.0306119)
Supplement: Attachment — Submitted filename: PLOSONE_Revision Letter_FINAL.docx.odt [file pone.0306119.s002.odt]

PONE-D-23-26287
Observers’ Motivated Sensitivity to Outgroup Actors’ Intent
PLOS ONE


	


Dear Dr. Rutar,

Thank you for the opportunity to resubmit our paper. We have made significant changes to the manuscript in response to the reviewers’ and your comments. In this letter, I describe in detail the changes we have made. In the manuscript, our changes are highlighted in yellow. Your comments or the reviewers’ comments are written in italics. Our response is written in un-italicized font.


Many thanks,
William Staples
Jason Plaks
Response to the Editor:
1. Collect more data or reframe the paper in line with Reviewer 1's recommendation.
With a good deal of new text throughout the manuscript, we have significantly reframed the paper to address Reviewer 1’s question about whether the effects are generic ingroup/outgroup effects or effects that are specific to the groups in question. We have been persuaded to reframe the effect as one about stereotypes about specific stigmatized groups - African Americans, Conservatives, and Liberals - rather than a general ingroup/outgroup effect. We have also changed the title of the paper so that it no longer refers to ‘outgroups’, but instead to specific stigmatized groups. We now offer more measured, modest claims about what our data do and do not imply.

2. Correct the error pointed out by Reviewer 1 in their point 2 (having to do with the mismatch between the presented scenario and the appendix description).
We have now done so.

3. Take heed of Reviewer 1's point 3 about sampling.
We have done so. I provide more specifics below in the section that responds to Reviewer 1.

4. Explain and correct the error mentioned by Reviewer 1's point 4 (truncation of the horizontal axis).
We have now done so.

5. Reviewer 2 has raised serious concerns with your handling of race and racial stereotypes. Your response to their critique should be very thorough and detailed.
We have added a significant amount of new text throughout the manuscript that addresses these concerns. We provide more detail below in our response to Reviewer 2. But to briefly preview, we suggest that most of Reviewer 2’s concerns about how we operationalized race, though legitimate and worthy, require responses that are well beyond the scope of this paper. That is because this is not a paper about race and racism. It is a paper about judgments of intent. We simply used the actor’s race - in one study - as a device to manipulate participants’ interpretation of the actor’s action. This manipulation theoretically could have been done in any number of ways (for example, by varying participants’ financial incentives). The nuanced and complex issues about intersectionality and sociocultural influences that the reviewer raises are important areas of study but are tangential to our primary focus.

6. Address Reviewer 2's point about obsolete data.
As we note below, this criticism is more relevant to a paper that purports to be a detailed examination of the psychology of racism in 2024. Our paper does not have that goal.  Instead, as noted above, our race manipulation in one study was a means to an end, not an end unto itself. 

7. Both reviewers raise concerns about the ecological validity of your conclusions. That should be remedied (i.e., explained as a limitation) and you should provide a thorough explanation (to the reviewers) of why your data/conclusions are limited in this way yet still important.
We have added a lengthy new section to the General Discussion (pp. 28-29) that more clearly identifies ecological validity concerns and explains that we constructed our scenarios with the full understanding of their limited ecological validity. We go on to argue that this approach is often used in both philosophy and psychological research because such scenarios may help to identify and isolate specific, fundamental building blocks of moral cognition.

8. Thoroughly address Reviewer 2's point about ethical concerns (in their point 7).
 The reviewer writes, “Utilising race as a variable in scenarios of moral transgression without a nuanced or critical understanding of racial dynamics can inadvertently reinforce stereotypes.”
We respond to this point in detail below, but to foreshadow, we remind the reviewer that our studies received approval from our university’s rigorous Research Ethics Board (REB) - as have presumably all published papers that in the recent history of social psychology that have used similar manipulations of a target person’s race. We would humbly suggest that it is the job of such ethics review boards (composed of experts in scientific ethics) to make such ethical judgments. We received REB approval for these studies and have submitted the requisite evidence to PLOS One when we submitted the manuscript. In other words, an impartial body has determined that the risk of inadvertently reinforcing stereotypes is negligible and is outweighed by the potential scientific benefit.

Response to Reviewer #1: 
Major comments:
1. In the main text, the scenario in Study 1 is about Alex trying to kill Linda by drowning. In the appendix the scenario is about JG trying to kill his uncle by running him over with his car.
Thank you for pointing out this oversight. It has now been corrected.

2. The actors in Study 1 are either Black or White men. The Mturk sample hardly includes Black participants. Would the results differ for Black participants assessing the intentionality of Black/White actors? If not, it would suggest that the results are not about the actor belonging to an outgroup, but about the actor being black. This is a crucial point, because the paper is about “outgroup actors”.
We have changed the text throughout (pp. 7,8,14,17,18,24,26,27) so that it refers to conclusions regarding participants’ judgments of the Black actor, the conservative actor, the liberal actor, or ‘members of stigmatized groups’ (rather than using the language of ‘ingroup/outgroup’).

3. The ABPC scale goes from 1 to 7. In Figure 5, which presents the distribution of ABPC scores, the horizontal axis terminates at 5, giving the *false* impression that the “conservative” tertile is somewhat above the center of the scale (i.e., is somewhat conservative). But in reality, there are hardly any participants with an ABPC higher than 4, suggesting that the “conservative” tertile is actually more liberal than conservative.
Our implementation of the ABPC used a scale with a range of 1-5. The ABPC in the appendix is the original ABPC, differing only in its use of a scale with a range of 1-7. Nonetheless, we have now changed our language to read ‘relatively conservative’ and ‘relatively liberal’. We now acknowledge even more explicitly in the text that the distribution of political orientation scores did not use the entire scale. Nonetheless there was enough variability in the data to allow clear patterns to emerge. As we now state on p. 23-24, this means that the effects we report may, in fact, underestimate the true magnitude of the difference between liberal and conservative participants.

Minor comments:
1. Add page and line numbers
We have now done so.

2. “In other words, motivated cognition is often most evident in instances when ambiguity provides plausible deniability, or the illusion of objectivity (e.g., 23)” – not clear, sentence seems out of place.
We have added additional text and citations on pp. 14-15 explaining that this is a canonical principle of motivated cognition. Decades of studies indicate that people generally only engage in motivated cognition when there is little danger of being exposed as biased or delusional.

3. Add error bars to figures
We have now done so.

4. The figures would be easier to understand if arranged in a 2x2 table, with row labeled “High Distal Intent”/”Low Distal Intent”, and columns labeled “High Proximate Intent”/”Low Proximate Intent”
We have now arranged the bar graphs into the 2x2 table format you suggest.

5. “Second, political intergroup bias may be stronger than racial intergroup bias” – this seems too general. It is based on the current evidence about *moral* reasoning and attribution. Since political groups are plausible construed as based on moral differences, it may be that other forms of bias—not/less related to moral issues—will not display a similar pattern.
We are persuaded by the reviewer. We have changed the sentence to read “political intergroup may be considered more morally justified than racial intergroup bias”.

6. Homicide is a very specific and extreme moral violation, which is likely not relevant to most people’s daily lives. I find it hard to draw general conclusions from this scenario.
We have added additional text on p. 29 noting that the results of these studies may vary if the actor committed, for example, a white-collar crime.

Response to Reviewer #2: 

1) Conceptual Superficiality and Gradation of Intentionality
The manuscript's exploration of intentionality, particularly the proximal and distal intent framework (PIDI), attempts to offer a nuanced understanding of how intentions are perceived and judged. However, this exploration falls short of a truly gradational analysis, failing to convincingly argue for the distinctiveness and utility of the PIDI framework beyond existing theories in moral philosophy. The delineation between proximal and distal intent, while intended to enrich the understanding of intentionality, is not clearly or convincingly distinguished from pre-existing theories, which already address the complexity of intentions and their moral evaluations.
We very much appreciate the reviewer’s exhortation to argue convincingly that the PIDI is conceptually distinct from previous models. Unfortunately, the reviewer does not name a single example of a pre-existing psychological theory that might contain excessive overlap with ours. Nonetheless, this comment spurred us to examine the psychology literature further. On pp. 5-6, we have enriched the literature review with several additional pre-existing accounts of laypeople’s theories of intentionality. We spell out more explicitly how our approach differs from (and builds upon) others, especially the influential Malle & Knobe (1997) model. For instance, on p. 6, we now write:
We view the PIDI framework as extending several of Malle and Knobe’s elements by	introducing proximal versus distal variants. For example, Malle and Knobe
operationalized intention as “intending, meaning, deciding, choosing, or planning to
perform the act” (p. 107). The PIDI model asks, what is the actor’s subjective
representation of the ‘act’? For example, does he think he is ‘pulling a trigger’ or
‘murdering his uncle’? Similarly, consider the concept of “desire.” Malle and Knobe
(1997) defined desire as “a wish for a particular outcome” (p. 106). The PIDI model asks,
what exact desire is forefront in the actor’s mind, pulling the trigger or killing the uncle?
By now, numerous studies have provided evidence that perceivers are quite sensitive to
these distinctions (15,16,18). 


Such judgements of intent also form the basis of most legal systems and sentencing, yet no reference is made to the vast body of research in criminology or criminal psychology.
We had initially excluded criminology and legal theory citations in order to keep the paper more focused on the psychological literature, but we have now re-inserted a range of relevant legal and philosophical works (e.g., pp. 27-28) that have, in fact, significantly informed our thinking. As now noted in the text, we have been particularly influenced by the writings of legal theorists RA Duff and HLA Hart. These philosophical theories of mens rea, however, have rarely been put to the empirical test. That is what our research program sets out to address (not only in this paper, but several others; see Plaks & Robinson, 2017 for an extensive review).

While this bidimensional approach is a commendable attempt to refine our understanding of how intention is perceived, the manipulations of proximal intent often come across as convoluted, and while the model promises a greater granularity and gradation in exploring the perceptions of intent, it only offers four categorical options, hence missing out on the promise of a more nuanced, dimensional model or exploring the possible variations of intentionality within the set categories.
We now state even more explicitly that we view PI and DI as continuous dimensions.  For example, on pp. 4-5, we now state:
It is important to note that we consider the presence or absence of proximal intent and
distal intent as continua, not dichotomies. Thus, at a given moment, an actor’s distal
intent may be more prominently in mind than her proximal intent, but that does not imply
that the proximal intent is absent.

In the present studies, we operationalize proximal and distal intent in a dichotomous fashion to permit the manipulation of both in a standard, social psychological 2X2 full factorial design. Indeed, whereas other psychological theories of intent often dichotomize intent (e.g., Schein & Gray, 2018), we explicitly do NOT view these dimensions as binary constructs. We are very clear about this in several places, including pp. 4, 29. Consistent with over 80 years of social psychological research, we use a 2X2 design with the understanding that doing so is a model of reality - not reality itself.
There is also no mention of the possible plurality of "distal" intents, which may converge or conflict in a situation ("proximal" intents seem entirely anchored in action and thus sequential).
Thanks to the reviewer’s astute comment, we now highlight that, for example, when J.G. presses the accelerator in the Proximal Intent Higher condition, this is a case when a second, different distal intent (avoid hitting pedestrian) has precedence in the actor’s mind - rather than the morally/legally-relevant distal intent (kill his uncle) (p. 5). On p. 28-29, we have added new text describing how the famous legal case of Oscar Pistorius represents an example of an actor who claimed to be acting with full proximal intent (pull trigger with awareness and control), but with a distal intent (kill intruder) that was different from the distal intent with which he was charged (kill girlfriend). We thank the reviewer for encouraging us to clarify that when we say, “Distal Intent Low” that is actually shorthand for “focal Distal Intent Low”.

Finally, the claim that "proximal" and "distal" intents are completely independent remains questionable.  
We agree. We never state that they are completely independent and do not wish the reader to infer that they are completely independent. We have added language such as “somewhat independent” and “partially independent” throughout the paper (e.g., pp. 4,7,27).  It is worth noting that in these studies (as well as virtually every other study we have ever run using the PIDI framework (N > 15,000), the data repeatedly reveal two main effects (DI, PI), but no DIxPI interaction. This indicates that, empirically speaking, PI and DI do enjoy a significant degree of independence.


2) Insensitive Handling of Race and Stereotypes
The study's approach to racial dynamics, particularly through the framing of racial group membership in Study 1, raises concerns. Utilising race as a variable in scenarios of moral transgression without a nuanced or critical understanding of racial dynamics can inadvertently reinforce stereotypes.
We would respectfully suggest that, for decades, experimental social psychologists have purposely created simplified, operational models of reality to manipulate their variables of interest in specific studies. But the creators of such studies typically maintain the full understanding that these are simplified models. Importantly, we would also note that countless institutional ethics review committees (including our own) have determined that the likelihood that participants will become more racist by participating in a study that varies the actor’s race is negligible and outweighed by the potential scientific insights. As per PLOS ONE policies, we have furnished our institutional ethics review approval. This board of experts in ethics and law has scrutinized our proposal, considered the possible harm to participants, and decided that any potential harm falls within the latitude of everyday experience. To learn more about the ethics review process at our university, please see: https://research.utoronto.ca/ethics-human-research/research-ethics-boards.

 By relying on racial stereotypes and dated instruments (such as the use of the Attitudes Toward Blacks Scale (ATBS), which was initially developed in the 1970s as “Attitude Toward Negroes Scale”)
We would respectfully suggest that we did not ‘rely’ on the ATBS scale. In fact, we barely discuss it at all. It was included as an afterthought merely to test for possible moderation of our basic effect…and, in fact, it yielded no significant effects.

and the assumptions that South Asian participants will identify with "the White ingroup" and judge Black people as "outgroup members", the study inadvertently reinforces simplistic and monolithic views of racial groups, failing to acknowledge the complex, intersectional identities and experiences of individuals within these groups.
The reviewer is correct to suggest that it is unfair and prejudicial to assume that non-Black persons of colour use White socio-racial schemata. We thank the reviewer for their vigilance on this topic, especially given that it was a concern for us as well. To address this concern, we made sure to test for effects between demographic subsamples. These tests revealed no significant differences between South Asian participants and White participants.
 Further, we would add that we agree with the reviewer’s other point. Issues of intersectionality are indeed vital and critical areas of study. However, ours is a paper on a different topic:  lay theories of intentionality. This paper was never intended to be a thorough investigation of race perception. That is why we conducted Study 2, which has nothing to do with race. A nuanced and complex investigation of intersectionality is a valuable and important topic, but it falls well beyond the scope of this paper.  

This oversight is additionally evident in the failure to include Black people as participants in Study 1, hence always positioning White (or non-Black, which is often conflated with White, and sometimes used to include Asian participants) participants as the judges of the behaviour of Black (or White) actors. Furthermore, this reinforces the default White gaze, and does not explore the possible biases in moral judgements of White and Black actors from the perspective of Black participants.
We have added new text that discusses these points on pp. 16 and 27. Our inclusion of Study 2, demonstrates that our concern in this paper was not with race, per se. Our main interest was to capture observers’ differing thresholds for intent applied to stigmatized (vs. non-stigmatized) actors - however that stigma is operationalized.

It is also interesting that in a scenario designed to test the differential perceptions of intent on the basis of racial identity, the racial identity of the character Linda is never disclosed. That of course does not mean that participants imagined her as raceless, and what identity they attributed to her might have affected the severity of their judgements in various ways.
We did not manipulate Linda’s race because it would create an unwieldy design, doubling the amount of participants needed to capture a possible 4-way interaction:  i.e. a  2 (Proximal Intent: high vs. low) x 2 (Distal Intent:  high vs.  low) x 2 (Actor Race:  Black vs. White) x 2 (Victim Race:  Black vs. white) full factorial design. We can, however, envision future studies that also manipulate the victim’s race. We have added new text on p. 16 that addresses these interesting questions.
Finally, the data is 10 years old, and the last decade has been pivotal for the changing landscape of racial relations in North America (the last term of Obama's presidency, Trump's presidency, Charlottesville Rally, Ferguson, the Black Lives Matter movement, etc.). The paper never takes the effort to contextualise racial attitudes in the broader sociocultural and historical context, treating them as purely individual variables.
We thank the reviewer for this point. The reviewer seems to be implying that non-Black participants in 2014 were more motivated to disparage Black actors than non-Black participants are today. This claim is contestable. Recent data clearly indicate that support for Black Lives Matter has dropped precipitously since 2020 (Pew Research Center, June 2023). In any case, even if the reviewer is correct and non-Black participants in 2014 were more motivated to disparage Black actors, this is tangential to our aim. Our paper is about sliding thresholds in judgments of intent. Nonetheless, we have added new text on p. 16 that notes that the results might differ if re-tested today.
Regarding the reviewer’s objection to treating racial attitudes as ‘individual variables’:  We would respectfully respond that this is a psychology paper, not a sociology paper. In psychology, the individual is the unit of analysis, not macro-level structures or systems. The introduction of such variables would break through the bounds of the discipline in a manner that, as experimental psychologists, we feel unqualified to pursue.

…on page 14, the authors assert that "A voluminous psychological literature has demonstrated that people display a range of motivated and non-motivated biases when processing information about racial outgroups, especially young, Black men (e.g., 24-26)" – it is unclear whether there is the greatest body of research on the biases towards young, Black men, or whether the research shows the greatest extent of biases targeting young, Black men.
On p. 9, with new text we clarify that both are true.

…there is no information about whether this relates to a criminal setting (as in the study) or other settings (research suggests greater biases against Black women in workplace and healthcare settings, in judgements of attractiveness, in social and economic marginalisation, etc.; not to mention other intersecting axes of marginalisation, such as disability, class, education, ...).
Again, these are great questions for a different paper - one that is squarely focused on delving into the complexities of race perception in different contexts, rather than a paper that is focused on lay theories of intent.

This is just an example of the way that the authors use quick and convenient statements to justify their methodological choices (showing a picture of a young Black/White actor to explore the differences in perceptions of intent) without acknowledging context, intersectionality, or cultural and narrative templates circulating around racial criminal stereotypes (especially in North America). And while contemporary (liberal) racism tends to operate along quite subtle lines, the authors regurgitate 18th century level discourse about the mental capacities of Black people (e.g., “Study 1 data suggest that non-Black participants assume that Black actors possess the capacity to hold a broader aim (distal intent) in mind.” (p. 20)). While the authors pose the hypothesis on the basis that "Black individuals are more strongly governed by immediate, impulsive concerns, rather than abstract, rational aims (27-29)" (p. 14), attributing such deeply racist beliefs to their hypothetical participants, the repetition of such claims in scientific literature can serve to validate and reinforce these highly problematic views.
Unfortunately, there is considerable evidence from numerous highly-cited papers on race perception in social psychology that non-Black participants often do dehumanize Black actors in a variety of ways (e.g., Goff et al., 2008; Williams & Eberhardt, 2008; Bonam et al., 2016; Cheung, Schmalor, & Heine,  2021). The data speak for themselves; 18th century ideas continue to persist in 21st century American minds (as much as we may wish it to be otherwise). Thus, the dehumanization hypothesis was not an unreasonable a priori hypothesis. 
It is worth noting, however, that this hypothesis was not supported by the data. We view this as good news and a potential sign of evolving views of race over time. Additional good news: There was no significant main effect of target race, indicating that participants did not, in the aggregate across all conditions, judge the Black actor more harshly than the White actor. Instead, participants judged the Black actor moderately more harshly than the White actor (𝛃 = -.248) in only one of the four conditions.

3) Theoretical Contributions and Distinctions from Existing Theories
The paper struggles to articulate the unique contributions of the PIDI framework and its distinction from existing moral psychological and philosophical theories.
Although the reviewer does not cite one existing psychological account of how laypeople mentally represent intentionality, we have taken this exhortation to heart. We have now expanded our review of the literature to include several additional classic and contemporary accounts of observers’ models of intent that were not in the previous draft.  We explain in each case how our model is different. In a previous review paper (Plaks & Robinson, 2017), we provided a fuller treatment of where the PIDI model stands in relation to the existing moral and social psychology literature. 

The notion that global or overarching motives (distal intent) are considered secondary to specific actions (proximal intent) in moral judgments is not adequately justified or explored, leading to confusion about the framework's theoretical underpinnings and its practical implications for understanding moral responsibility.
At no point in the paper do we state that overarching motives are secondary. Indeed, the PIDI model states that proximal intent and distal intent are two equal competitors in laypeople’s minds.


4) Sensitivity to Context and Narrative Construction
The construction of scenarios for judging moral transgressions lacks justification and sensitivity to broader social and cultural contexts. By relying on questionable narrative templates and stereotypes (such as men resorting to extreme physical violence against women, women scheming against husbands and committing premeditated murder), the study overlooks the rich tapestry of moral reasoning that individuals employ, which is shaped by diverse social, cultural, and personal experiences.
We agree that such scenarios fail to account for the ‘rich tapestry of reasoning that individuals employ’. However, we pursued a common research strategy in experimental psychology, and moral psychology in particular. Like most other researchers in the history of the field, we are not attempting to account for the rich tapestry of moral cognition in one study. Instead, we are using purposefully contrived scenarios to isolate one small piece of the puzzle. By analogy, consider that the Trolley Problem, which has been used in countless, influential papers in moral psychology for the past 20 years, certainly lacks ecological validity. But researchers continue to use such scenarios because they are convinced by the evidence that it captures a fundamental moral tension that everyone must navigate every day (i.e. How much wrong is justified in the name of the greater good?). Similarly, in perceptual psychology, researchers often present participants with stimuli that purposely lack ecological validity (e.g., random squares of color; various shapes and lines). But they do so because these data may provide insights into the building blocks for more complex perception and cognition. Our approach is similar. We are trying to identify building blocks that form only a small part of individuals’ more complex moral cognition. We have added a new section of the paper (pp. 29-30) that presents these ideas. 

5) Biases and Assumptions
The study's hypotheses and interpretations are heavily influenced by selective previous research and assumptions that may not allow for alternative interpretations of the data. Especially the framing of hypotheses in Study 1 is very problematic; they rely on cherry-picked previous research and act as templates for interpretation, whereas numerous other hypotheses could be made, and the data could be explained with other, competing theories.
This comment spurred us to enrich our literature review. We have identified five previous psychological accounts that attempt to characterize laypeople’s theories about what makes an act intentional:  Ossorio & Davis (1968), Shaver (1985), Malle & Knobe (1997), Laurent et al. (2015), and Levine & Leslie (2023).  With more detail than in the previous draft, we describe on pp. 5-6 how the PIDI is different from - and builds upon - each of these previous accounts.
Moreover, because the proximal–distal distinction in intentionality is in many cases questionable, and because the quantitative data does not illuminate the underlying psychological mechanisms, the interpretation and discussion often seem dubious.
We sincerely welcome concrete feedback. Unfortunately, the reviewer never articulates exactly what s/he finds questionable or dubious about the proximal-distal distinction. This vagueness makes this critique difficult to respond to.

The biases and assumptions are also evident in the framing of moral judgments from the perspective of predominantly white or non-black participants, without adequately considering the perspectives of other racial groups or exploring the potential for motivated racial moral reasoning beyond simplistic binaries.
We address this issue above.


6) Political Orientation Conceptualisation:
The study’s conceptualisation of political orientation as a binary — distinguishing only between liberals and conservatives — oversimplifies the complexity of political identities
We measured political orientation as a continuous variable. We divided the sample into two groups because the distribution was clearly bimodal (see Figure 5) and there is, by now, enormous evidence that ordinary North Americans increasingly view political identity in polarized, binary terms - and not in the nuanced way that the reviewer envisions. Just a few of the citations reporting massive increases in polarization over the past 20 years:  Mason, 2018; Iyengar et al., 2019; Abramowitz, 2018.
Political scientists have dealt with the issue raised by the reviewer by articulating the concepts of ‘symbolic’ and ‘operational’ political identity (Ellis & Stimson, 2012). We have added text that makes the difference between the two apparent; specifying that our measurement of political orientation is done with the ABPC and therefore, a measurement of operational political identity.

This binary is notably North American-centric and fails to account for the increasing diversity within the political spectrum, even within North America itself.
We agree that the paper is North American-centric and have added new text on p. 26 clarifying that North American political identity differs from political identity in other parts of the world. The reviewer’s claim that there is increasing diversity within the North American political spectrum is at odds with the evidence of increased polarization (see citations above). Nonetheless, even if the reviewer is correct, such variability in the data would only make it more difficult to obtain our effects. The fact that we reliably found that participants judged the anti-partisan actor more harshly than the partisan actor - despite the noise in the data that the reviewer assumes - suggests that our effects are quite robust.

Such an approach neglects rich literature from political psychology and political sciences that could provide nuanced insights into biases and stereotypes about political outgroup members. The lack of acknowledgment of this literature leaves the study with a significant conceptual gap, undermining the applicability of its findings to broader discussions on political ideology and moral judgment.
Just as Study 1 was not intended to provide a comprehensive account of the psychology of race, Study 2 was not intended to provide a comprehensive account of political psychology. Like race, the actor’s political identity was used merely as a device - as a means to an end. Our more modest focus was on how people judge an actor who committed a homicide by accident. Our aim was to find ways to coax participants to give the actor more versus less benefit of the doubt. In both studies, we succeeded in doing that.
7) Methodological Concerns, Ecological Validity, and Future Directions
The lack of information on the briefing and debriefing procedures, especially given the studies' sensitive nature, raises ethical concerns.
This information was included in the Ethical Statement on the cover page of the manuscript. 

There is also a lack of commentary on how the application of scales assessing the participants' racial attitudes and political orientation might have acted as priming and biased the subsequent results.
In Study 1, there were no significant effects involving the measures of racial attitudes. As such, there is no evidence that they affected participants’ responses. In Study 2, The ABPC was presented after participants viewed the stimuli and responded to the dependent variable, meaning that the measurement of political orientation could not have biased responses. Of course, one could argue that the presentation of the stimuli may have biased responses on the ABPC. This is highly unlikely as the stimuli use subtextual indicators of the actor’s symbolic political identity, while the ABPC prompts participants on their personal operational political identity. The risk of biased responding to the ABPC is small, indeed, smaller than the risk of biasing responses to our dependent variable had we presented the ABPC before our stimuli. It is for this reason that the ABPC was placed after the dependent variable. This order presents the lowest risk of any one component of our study contaminating others.

The claim that the study provides “a standardized, theoretically – and empirically – grounded template for future researchers who wish to measure intergroup judgments involving ambiguously-intentional acts” (p. 32) appears overstated given the limitations of the study's ecological validity and sensitivity to context. The assertion of offering a standardised template for future research is premature, especially considering the unexamined assumptions in the process of "standardisation" and the lack of in-depth study and understanding of the processes of moral reasoning that the PIDI framework purports to illuminate.
We are persuaded by the reviewer. We have removed that text altogether. Elsewhere, we have significantly altered the text to scale back our claims.

There is also a lack of discussion on potential applications of the findings, such as understanding biases and judgments in criminal justice contexts, where the perceptions and biases of jury members can have severe effects on people's lives.
We have added an extended section on p. 29 that accomplishes this.

Finally, the paper's theoretical and methodological foundations would benefit from greater diversity among the authors and participants, allowing for a broader and more nuanced exploration of the topics at hand.

We now note this in several places throughout.

References
Abramowitz, A., & McCoy, J. (2019). United States: Racial Resentment, Negative Partisanship, and Polarization in Trump’s America. The ANNALS of the American Academy of Political and Social Science, 681(1), 137-156.
Ellis, C., & Stimson, J. A. (2012). Ideology in America. Cambridge University Press.
Iyengar, S., Lelkes, Y., Levendusky, M., Malhotra, N. & Westwood, S. (2019). The Origins and Consequences of Affective Polarization in the United States. Annual Review of Political Science, 22, 129-146.
Mason, L. (2018).  Uncivil agreement: How politics became our identity. Chicago:  University of Chicago Press.
