# Supplementary material for: Observers’ motivated sensitivity to stigmatized actors’ intent
Source: PLoS One. 2024 Sep 6;19(9):e0306119. doi: 10.1371/journal.pone.0306119 (PMC11379140; doi:10.1371/journal.pone.0306119)
Supplement: Attachment — Submitted filename: Staples_Plaks_Review.docx [file pone.0306119.s003.docx]

**Review**

The manuscript *Observers’ Motivated Sensitivity to Stigmatized Actors’ Intent* presents an interesting and relevant empirical investigation of the effects of racial and political stigma on observers' judgments of intentionality and moral culpability. The authors effectively employ a robust methodological framework, manipulating both proximal and distal intents in hypothetical scenarios of homicide across two studies. They show that actors from stigmatized groups are judged more harshly than their non-stigmatized counterparts when the intent of the individual from the stigmatized group is ambiguous (Study 1) or distal intent is absent (Study 2). Their results build on existing work by demonstrating the usefulness of distinguishing between two (at least partially independent) forms of intent. This demonstrates a significant contribution to the literature on motivated cognition and lay theories of intentionality.

The authors have thoroughly responded to revisions suggested by the previous reviewers. This is especially apparent from the following cases.

- The problem of ecological validity was further elaborated. The authors clarify that despite the potentially limited ecological validity of such experimental studies, they can help to identify and isolate specific, fundamental building blocks of moral cognition.
- The authors explain the paper's original contribution to existing psychological work on intentionality lays by manipulating the presence/absence of the two varieties of intent (proximal and distal) in studying bias in judgements of intent.
- As pointed out, varying the actor's race and political orientation is a standard way of experimental manipulation, meaning that a detailed analysis of psychology of racism is not necessary for this paper to achieve its aim.
- While a categorical distinction between proximal and distal intent does not reflect the continuous nature of intentionality, the authors acknowledge this at multiple points in the paper and explain the usefulness of this dichotomy in this study.

Most of the concerns raised in the previous round of review were addressed by expanding the substantive arguments, literature review and additionally acknowledging the study’s (several) limitations and open questions. The suggestions that were not directly incorporated in the paper were satisfactorily addressed in the authors’ response to reviewers (e.g., that their operationalization of race follows a standard approach in experimental papers and that an entirely different operationalization both exceeds the paper’s scope as well as is not necessary given the paper’s focus on judgements of intent).

The authors have addressed the key methodological shortcomings highlighted by Reviewer 2 to the extent that these issues can practically be resolved. While some of these limitations remain, such as the problem of limited ecological validity, the authors have demonstrated that their work aligns with the established standards of the scientific community in general, and the principles and scope of experimental psychology specifically. Additionally, the paper’s scope is limited to researching bias in judgements of intent and does not intend to be a thorough study on the psychology of racism, intersectionality and broader sociocultural factors affecting individual’s judgements.

In light of this, I suggest the paper be accepted with minor revisions.

Specific suggestions

- On page 7: it might not be entirely clear to the reader how the present study differs specifically from the several studies mentioned by the authors in which both forms of intent have been manipulated experimentally. A sentence at the end of the paragraph making the specific contribution of the present paper in this regard might be helpful.
- On page 9: “(…) compared to White individuals. Black individuals (…)” – unnecessary comma.
